# The Rash, the Weakness, and the Nephritis: Nerve and Kidney Biopsy Findings in Eosinophilic Granulomatosis With Polyangiitis

**DOI:** 10.7759/cureus.11676

**Published:** 2020-11-24

**Authors:** Mohd Elmugtaba Ibrahim, Anand Deonarine, Payton A Gore, Harvey R Lewis, Eduardo C Alvarado

**Affiliations:** 1 Internal Medicine, Howard University Hospital, Washington, DC, USA; 2 Internal Medicine, Howard University College of Medicine, Washington, DC, USA

**Keywords:** churg strauss, eosinophilic granulomatosis with polyangiitis

## Abstract

We present a case of eosinophilic granulomatosis with polyangiitis (EGPA) or Churg-Strauss syndrome in a 66-year Caucasian female who presented with a severe pruritic itch and a progressive upper and lower extremity weakness of unknown duration. The diagnosis of EGPA in this patient remained elusive for an extended period of time due to the absence of respiratory symptoms. In this article, we also discuss the histologic features of EGPA seen in biopsies of the kidney and the nerves and highlight the value they play in diagnosis.

## Introduction

Eosinophilic granulomatosis with polyangiitis (EGPA) (formerly known as Churg-Strauss syndrome) is a systemic, small to medium vessel vasculitis that affects a variety of organs and typically presents with asthma and eosinophilia as the hallmark of the disease. However, the disease symptomology occurs usually in three different phases at varying time intervals, which can make an initial diagnosis difficult and delay the treatment [1].

The biopsy of the affected organs is particularly useful in suspected cases with an unclear presentation. Histological examination of tissues affected by EGPA reveals tissue eosinophilia, necrotizing vasculitis, and eosinophil-rich granulomatosis. Commonly affected organs include the upper airway tract and lung involvement, peripheral neuropathy, cardiac, and skin lesions.

We would like to present a case in which biopsies of the nerve and kidney helped establish the diagnosis of EGPA in an adult patient who was misdiagnosed despite multiple hospitalizations.

## Case presentation

We present a case of a 66-year-old Caucasian female with a past medical history of hypertension, asthma, fibromyalgia, Hashimoto's thyroiditis, stroke with pseudobulbar palsy, irritable bowel syndrome, seizure disorder, peripheral vascular disease, bronchitis, hyperlipidemia, and chronic respiratory failure who presented with complaints of abdominal pain for two weeks, severe pruritic itch, and a progressive upper and lower extremity weakness of unknown duration. Of interest, the patient had no respiratory complaints at the time of presentation and the entire admission course.

Initial laboratory investigation revealed acute kidney injury and showed leukocytosis and eosinophilia of 6.4% that later peaked at 34.7%.

**Figure 1:**
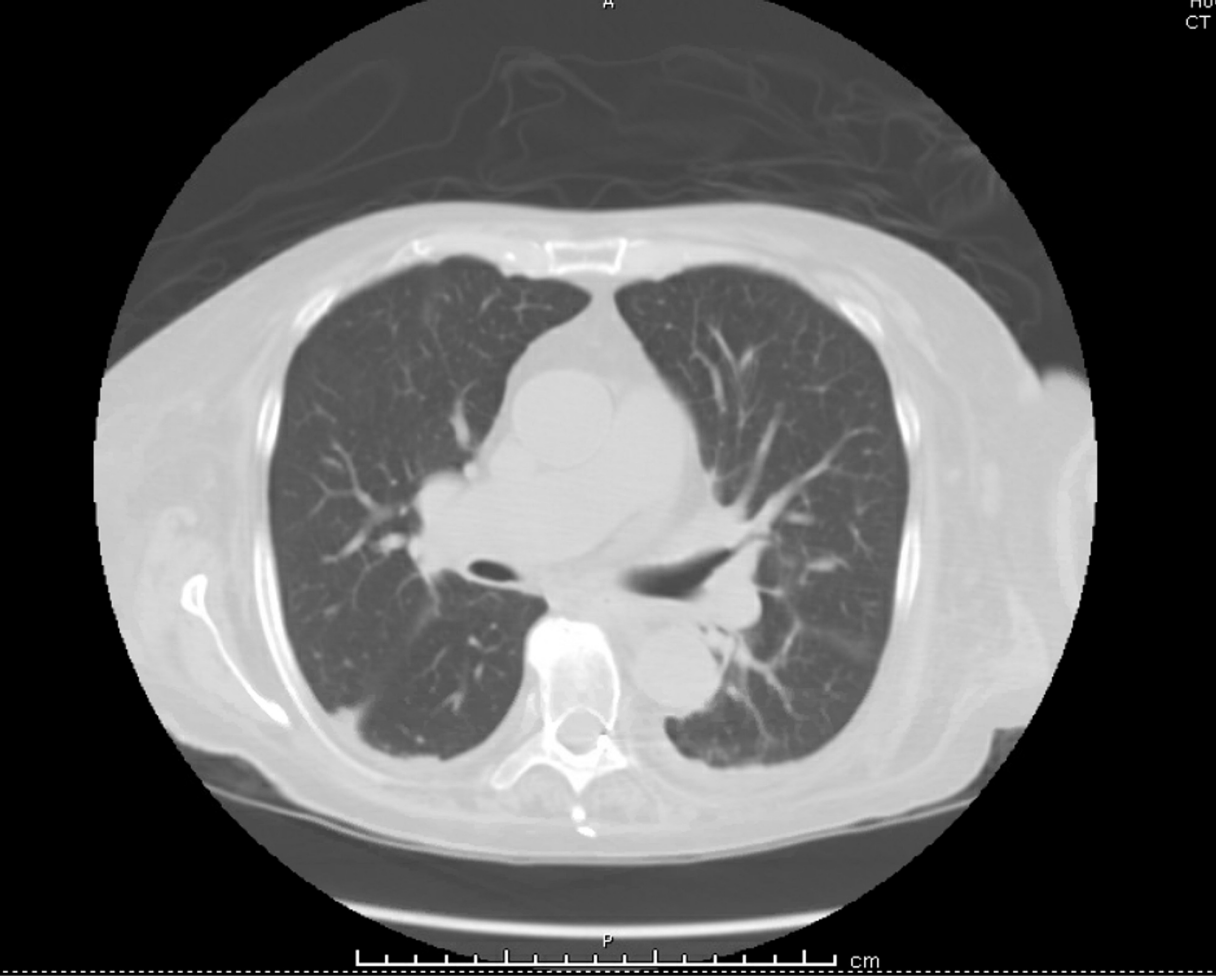
High-resolution CT of the chest reveals no evidence of parenchymal lung disease CT: computed tomography

The significant radiological finding included computed tomography (CT) of the head that revealed a porencephalic right lateral ventricular cyst. A high-resolution CT of the chest showed no evidence of interstitial lung disease, patchy opacities in both lungs, worse in lower lobes, small mediastinal, and hilar adenopathy (Figure 1).

The patient underwent a thorough rheumatologic investigation, which revealed antineutrophil cytoplasmic antibodies (ANCA) profile with weak positivity for C-ANCA, P-ANCA, and elevated myeloperoxidase antineutrophil cytoplasm (MPO 5) antibody and no antibodies for PR-3.

**Figure 2:**
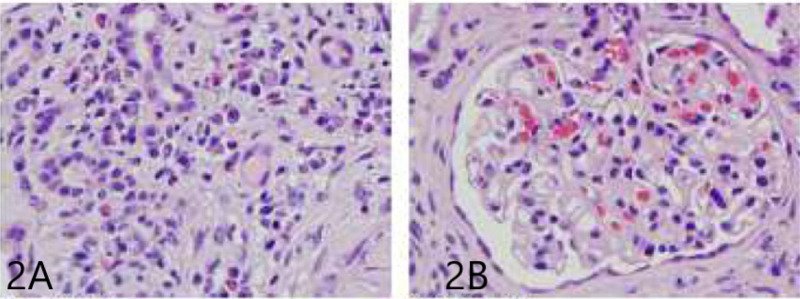
2A. H&E stain showing vascular sclerosis (arterial intima thickening), interstitial inflammation; 2B. H&E showing crescentic glomerulonephritis H&E: hematoxylin and eosin

The patient underwent a kidney biopsy to investigate the worsening renal failure, which revealed pauci-immune crescentic glomerulonephritis with fibrinoid necrosis and positivity for P-ANCA. Approximately 56% of glomeruli were globally sclerotic or approaching global sclerosis (based on light microscopy and immunofluorescence microscopy), vascular sclerosis (arterial intima thickening), interstitial inflammation (lymphocytes, plasma cells, and eosinophils), evidence of acute tubular injury, and mild interstitial fibrosis. See Figure 2.

A nerve conduction study of the ulnar and radial nerves revealed findings suggestive of myopathy, more prominent distally. This was followed by a sural nerve and a muscle biopsy of the right leg, which showed dropout of myelinated axons, segmental demyelination, and axonal degeneration. The muscle biopsy showed neurogenic atrophy with scattered lymphocytes and upregulation of major histocompatibility complex (MHC) suggestive of autoimmune etiology.

A diagnosis of EGPA was made based on the clinical, laboratory, and biopsy findings, and intravenous (IV) steroid pulse therapy was initiated. The patient’s pruritis and kidney function dramatically improved, and eosinophilia resolved after pulse therapy with IV steroids. However, the upper and lower extremity weakness remained unchanged. The patient was discharged on oral steroids with outpatient rheumatology follow-up.

## Discussion

EGPA, formerly known as Churg-Strauss syndrome, may present between 14 and 75 years of age, with a mean onset range of 38 to 54 years [1]. However, pediatric studies have identified EGPA in children as young as 4 [2]. The mean age of diagnosis is approximately 50 years [2]. The incidence of EGPA is 1.3 to 6.8 per 1,000,000 patients per year, with an overall prevalence of 10.7 to 13 per 1,000,000 patients. Also, EGPA has no gender prevalence [3].

The pathogenesis of EGPA is complex: the disease often involves exposure to allergens or drugs, but an HLA-DRB4 genetic background has been recognized as a possible predisposing factor. T-cell helper cell (Th) 2 response is prominent, with upregulation of IL-4, IL-13, and IL-5. The activated eosinophils, as a result of the immune cascade, cause tissue damage by releasing their granule proteins. Prominent IgG4 and IgE responses point towards humoral immunity dysregulation as well [4].

Additionally, EGPA is known to be linked to P-ANCA, also known as MPO-ANCA. However, unlike other vasculitides such as granulomatosis with polyangiitis and microscopic polyangiitis, where the prevalence of ANCA is approximately 70%-95%, the prevalence of ANCA in Churg-Strauss is approximately 40% [5].

EGPA histopathology comprises tissue eosinophilia, necrotizing vasculitis, and extravascular eosinophilic granulomas. Fibrinoid necrosis and eosinophilic vessel wall infiltration are hallmarks of EGPA vasculitis. Granulomas involve the arteries, but the more EGPA‐specific lesion is the extravascular granuloma, which consists of a core of necrotic eosinophilic material surrounded by palisading lymphocytes and epithelioid and multinucleated giant cells [6].

EGPA typically develops through three phases:

1) The allergic phase (phase 1), distinguished by the occurrence of asthma, allergic rhinitis, and sinusitis [7]. In this particular case, the patient has a history of asthma but had no respiratory symptoms during her hospital stay, which is atypical as 95-100% present with respiratory symptoms [4]; 

2) The eosinophilic phase, in which the main pathological finding is the eosinophilic organ infiltrations (e.g., lungs, heart, and gastrointestinal system);

3) The vasculitic phase, characterized by purpura, peripheral neuropathy, and constitutional symptoms [7].

Of interest, in this case, is that there is kidney and nerve involvement. The vasculitic phase is typically preceded by constitutional symptoms (e.g., fever, weight loss, fatigue). Peripheral neuropathy is a key feature of this phase, affecting approximately 70% of the patients. The neuropathy is characterized by axonal damage on electrophysiological studies and frequently affects the peroneal, tibial, ulnar, and median nerves; the most common pattern is mono neuritis multiplex, often complicated by asymmetric foot or wrist drop, which may progress to symmetric or asymmetric polyneuropathy, as seen in this patient, and histology usually shows epineural lymphocytic vasculitis with rare eosinophils [8]. The nerve biopsy, in this case, did not show any evidence of vasculitis and was completely devoid of eosinophils.

Kidney involvement is present in approximately a third of the patients. The prevailing picture is ANCA-associated necrotizing crescentic glomerulonephritis; however, other forms of nephropathy also may occur [9]. These features were present in this particular case.

The diagnosis of EGPA based on American College of Rheumatology (ACR) classification criteria for Churg-Strauss syndrome is made by the presence of 4 or more of the following criteria: asthma, eosinophilia >10%, mononeuropathy or polyneuropathy nonfixed, pulmonary infiltrates abnormalities of paranasal sinuses and extravascular eosinophils on biopsy [10]. In this case, the diagnosis was done based on peripheral eosinophilia, polyneuropathy, imaging findings of the lung on CT and was strongly supported with positive P-ANCA and presence of MPO 5 antibody.

According to the European League Against Rheumatism (EULAR), a positive biopsy is strongly supportive of vasculitis. We recommend the procedure to assist in the diagnosis and further evaluation of patients suspected of having vasculitis and remains a gold standard for diagnosis. We believe this is particularly important in cases where features of the disease are atypical like in this case and when end-organ damage is present [11].

The differential diagnosis of EGPA is wide and has overlapping features with several conditions such as hypereosinophilic syndrome, granulomatosis with polyangiitis, temporal arteritis, Takayasu arteritis, seronegative spondyloarthropathy, polyarteritis nodosa, microscopic polyangiitis, chronic eosinophilic pneumonia, hypersensitivity vasculitis, rheumatoid arthritis, sarcoidosis, tuberculosis, antiphospholipid antibody syndrome, and thrombotic thrombocytopenic purpura [12-13].

Medication choice for EGPA depends on the activity and extent of the disease, determined by a five-factor score (FFS) used to assess prognosis. The five-factor score includes proteinuria >1 g/d, renal insufficiency (stabilized peak creatinine 140 Kmol/L), cardiomyopathy, severe gastrointestinal manifestations, and central nervous system (CNS) involvement [14]. With FFS >1, glucocorticoids and cyclophosphamide are indicated. In patients with an FFS score of zero, patients may receive glucocorticoids alone. For patients who require unacceptably high glucocorticoid use, other immunosuppressants, such as methotrexate or azathioprine, may be added. Other additional treatments include intravenous immunoglobulins, rituximab, anti-IL-5 antibody, and mepolizumab. In this case, the patient FFS was >1 but due to a dramatic response to pulse steroid therapy, the use of cyclophosphamide was adjourned.

For FFS = 0, 1, and >2, the respective 5-year mortality rates were: 12%, 26%, and 46%, respectively [14].

## Conclusions

In conclusion, EGPA is a complex systemic disease that varies in presentation depending on the natural evolution of the disease. Histological evidence of involvement by eosinophilic infiltrates is of great utility to diagnose a treatable and, to some extent, reversible disease. This is especially true in the vasculitic phase of the disease where multi end-organ damage has occurred and the classic asthma symptoms are not present.
